# Study Protocol: A Pilot Study to Determine the Safety and Efficacy of Induction-Therapy, De Novo MPA and Delayed mTOR-Inhibition in Liver Transplant Recipients with Impaired Renal Function. PATRON-Study

**DOI:** 10.1186/1471-2369-11-24

**Published:** 2010-09-14

**Authors:** Andreas A Schnitzbauer, Marcus N Scherer, Justine Rochon, Johannes Sothmann, Stefan A Farkas, Martin Loss, Edward K Geissler, Aiman Obed, Hans J Schlitt

**Affiliations:** 1 Department of Surgery, Regensburg University Hospital, Regensburg, Germany; 2 Center for Clinical Studies, Regensburg University Hospital, Regensburg, Germany

## Abstract

**Background:**

Patients undergoing liver transplantation with preexisting renal dysfunction are prone to further renal impairment with the early postoperative use of Calcineurin-inhibitors. However, there is only little scientific evidence for the safety and efficacy of de novo CNI free "bottom-up" regimens in patients with impaired renal function undergoing liver transplantation. This is a single-center study pilot-study (**PATRON07**) investigating safety and efficacy of CNI-free, "bottom-up" immunosuppressive (IS) strategy in patients undergoing liver transplantation (LT) with renal impairment prior to LT.

**Methods/Design:**

Patients older than 18 years with renal impairment at the time of liver transplantation eGFR < 50 ml/min and/or serum creatinine levels > 1.5 mg/dL will be included. Patients in will receive a CNI-free combination therapy (basiliximab, MMF, steroids and delayed Sirolimus). Primary endpoint is the incidence of steroid resistant acute rejection within the first 30 days after LT. The study is designed as prospective two-step trial requiring a maximum of 29 patients. In the first step, 9 patients will be included. If 8 or more patients show no signs of biopsy proven steroid resistant rejection, additional 20 patients will be included. If in the second step a total of 27 or more patients reach the primary endpoint the regimen is regarded to be safe and efficient.

**Discussion:**

If a CNI-free-"bottom-up" IS strategy is safe and effective, this may be an innovative concept in contrast to classic top-down strategies that could improve the patient short and long-time renal function as well as overall complications and survival after LT. The results of **PATRON07 **may be the basis for a large multicenter RCT investigating the new "bottom-up" immunosuppressive strategy in patients with poor renal function prior to LT.

http://www.clinicaltrials.gov-identifier: NCT00604357

## Background

### Medical Problem

Early renal dysfunction after liver transplantation (LT) is reported with a frequency as high as 50% [1,2]. The introduction of the MELD-based allocation system in the Eurotransplant area in December 2006 led to an increase of the proportion of liver transplant recipients with renal dysfunction at the time of transplantation, since creatinine became a key component for the allocation of liver allografts [3]. Major risk factors associated with early posttransplant renal impairment are: preexisting diabetes mellitus, time on the waiting list with end-stage hepatic disease, application of blood products, liver allograft dysfunction and toxicity of Calcineurin-inhibitors (CNI) [4-11]. The risk of developing chronic renal failure after LT is approximately 20% after 5 years, associated with the use of CNI and a 4-fold increased mortality risk[12] - and these are data from the pre-MELD era. An additional problem in this specific patient group (impaired renal function at the time of LT and MELD-scores of 25 and/or higher), is a high risk for developing infectious complications [13]. Studies indicate that early infections are present in almost 85% of all patients, and become the most common cause of death early after transplantation. Notably, two-third of infections in liver transplant patients occur within the first 3 months after transplantation with a very high percentage (67%) of severe infections [14,15]. In general, the inflammatory response associated with infection is impaired by immunosuppressive drugs. This disturbed regulation increases the susceptibility for a broad range of normal and of opportunistic infections [13]. Therefore, patients with high lab-MELD scores hypothetically should require a rather low amount of immunosuppressive (IS) drugs during the first days to weeks after transplantation, while they are in a state of SIRS (systemic inflammatory response syndrome)-like state [16,17].

### Current treatment options

Most patients undergoing liver transplantation are treated with de-novo immunosuppressive protocol based on a CNI (cyclosporine or tacrolimus) and one or two additional drugs like steroids, mycofenolate mofetil (MMF) or induction therapy with anti-thymocyte globulin or anti-CD25-monoclonal antibodies. Current paradigms are based on a top-down strategy which is based on initially high dose IS treatment with a reduction in case of side-effects or complications. One of the major adverse effects of CNI is nephrotoxicity, a deleterious complication for long-term survival and quality of life [13-15]. However, there are no IS approaches that concentrate on a complete avoidance or "bottom-up" strategy, delaying the introduction of CNI or an mTOR-inhibitor until the patient really does require additional IS (acute rejection). To date there is only one prospectively randomized trial that investigated the influence of delayed, low-dose IS with Tacrolimus and MMF in patients with normal renal function. In this trial patients did profit from an early avoidance of Tacrolimus with regards to renal function [18].

### Aim of the pilot trial

The primary aim of this study concept is **(I) **to evaluate, if a de novo CNI-free IS regimen in patients with preexisting renal impairment at the time-point of LT can be applied safely with regards to steroid resistant acute rejections within 30 days after LT. **(II) **The secondary aim is to provide a therapeutic strategy that can improve or at least preserve an impaired renal function. **(III) **In a next consecutive sequential step we plan to perform a prospectively randomized 3-armed pilot-study that compares "bottom-up" CNI-free and CNI-containing IS regimens after LT in patients with impaired renal function and high-MELD-scores at the time-point of LT which would mean a change in paradigm from "top-down" to "bottom-up" IS strategies in at least a high risk population undergoing LT.

Reducing the risk for severe perioperative renal dysfunction (requiring dialysis) can be beneficial for patients with impaired renal function prior LT. It is likely that the early avoidance and latter "bottom-up" introduction of CNI and mTOR-inhibitors (everolimus or sirolimus) also reduces the incidence of early infectious complications, occurrence of hypertension, post-transplant diabetes mellitus and maybe decreases the risk for post transplant malignancies, the leading cause of death in patients undergoing solid organ transplantation.

## Methods and Design

### General Considerations and Study Population

This is a single-center study concept consisting of a prospective single-armed two-step pilot-trial (**PATRON07**) investigating safety and feasibility of a CNI-free, "bottom-up" IS strategy in patients undergoing LT with renal impairment prior to LT. Patients will be followed-up for one year after transplantation each. Since there is no clinical evidence for this IS approach, we decided to first perform an uncontrolled pilot-study investigating the safety and feasibility of a completely CNI-free approach

### Objectives of the study concept

The objective of **PATRON07 **is to evaluate a de novo CNI-free immunosuppressive regimen based on induction therapy with anti-CD25 monoclonal anti- body, mycophenolate mofetil (MMF/MPA), and delayed mTOR-inhibition. The primary endpoint is defined as the incidence of steroid-resistant acute rejection within the first 30 days after liver transplantation. It reflects the early potential and feasibility of the proposed immunosuppressive regimen with regards to safety and efficacy. In a liver transplant setting an acute rejection can be controlled with steroid treatment and does not necessarily mean an irreversible damage to the liver. Secondary objectives include the incidence of acute rejection(s), the number and the timing of acute rejections per patient within the first year after transplantation. A critical secondary endpoint will be the development of renal function at 1 week, 1, 3, 6 and 12 months after liver transplantation. This includes information on the number of patients requiring renal replacement therapy and its duration. During follow-up of 1 year liver allograft function, infectious complications, treatment failures defined as introduction of CNIs as well as side-effects affecting the hematopoetic system, tolerability, impaired wound-healing, the incidence of hepatic artery thrombosis and mortality will be explicitly documented and investigated.

### Trial Population

The collective we are aiming at are patients older than 18 years with a preexisting renal impairment at the time of LT. Patients will be eligible if the eGFR < 50 ml/min (Cockroft-Gault) and/or their serum creatinine levels > 1.5 mg/dL.

### Eligibility

Inclusion and exclusion criteria for **PATRON07 **are shown in table 1. Patients will be screened consecutively on the liver waiting list at Regensburg University and asked for their willingness to participate in this trial.

**Table 1:** Key In- and Exclusion Criteria for PATRON07 and BUILT_01

Inclusion Criteria	Exclusion Criteria
Patients undergoing primary liver transplantation.	Patients with pre-transplant renal replacement therapy > 14 days.

Patients older than 18 years.	Patients with platelets < 50.000/nl.

eGFR < 50 ml/min at the time point of transplantation	Patients with triglycerides > 350 mg/dl and cholesterol > 300 mg/dl refractory to optimal medical treatment prior to initiation of therapy with mTOR inhibition.

Serum creatinine levels > 1.5 mg/dL at the time-point of transplantation	Multiple organ graft recipients.

Patients with eGFR < 50 ml/min at the time point of transplantation or Serum creatinine levels > 1.5 mg/dL at the time-point of transplantation	Patients with signs of a hepatic artery stenosis directly prior to initiation of therapy with Sirolimus.

	Patients with a psychological, familial, sociologic or geographic condition potentially hampering compliance with the study protocol and follow-up schedule.
	
	Patients under guardianship (e.g. individuals who are not able to freely give their informed consent).

### Consent

Patients on the waiting list meeting the inclusion and none of the exclusion criteria will be informed about the possibility of participating in either **PATRON07**. They will be fully informed about the trial, the procedures, risks, potential benefits and treatment alternatives as well as the study management, insurance and documentation of personal data with regard to meeting data protection legislation.

### Procedures of minimizing bias

**PATRON07 **is a non-controlled prospective trial at a single high volume liver transplant -center, which will reduce bias due to surgical learning effects.

### Interventions

#### Pre- and Intraoperative Data

Baseline data will be documented including demographics, medical history, current medication, metabolic, liver and renal function as well as history of renal treatment. Intraoperative data (warm and cold ischemic times, blood-loss, requirement for blood products, incision suture times) and donor data (age, sodium, gamma-GT, body-mass index, infectious status) will be also documented.

#### Treatment Regimen

**Figure 1:**
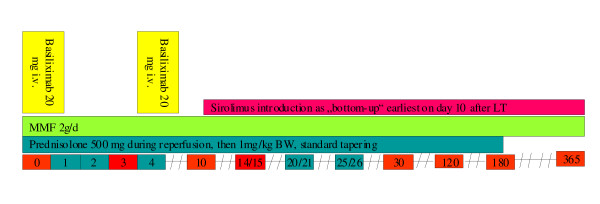
**IS treatment regimen for PATRON07**. Basiliximab will be applied on day 0 and 4 after transplantation as 20 mg i.v. application, MMF will be applied as 2 g/d bid, center-specific steroids will be applied and tapered by month 6 after LT, Sirolimus will no be started prior to day 10 after LT aiming at trough-levels of 4 to 10 ng/ml.

Prior to reperfusion 500 mg Prednisolone will be administered i.v. After the transplantation, a combination of anti-CD25-mAB (*basiliximab 20 mg on day 0 and day 4 after the procedure*), and MMF *2 g/d*, *2 applications per day i.v., later conversion to oral intake*) will be applied. On day 10 after LT *Sirolimus *will be introduced with a loading dose of 5 mg/d. Thereafter a dosage of 2 mg/d will be administered, aiming at 24 hours trough-levels for *Sirolimus *between 4 and 8 ng/mL. Steroids will be started on day 1 after transplantation with 1 mg/kg BW and will be tapered every 2 days for 5 mg to a dosage of 20 mg and for 2.5 mg every two days to 7.5 mg. Thereafter the dosage will be reduced to 5 mg and 2.5 mg for 1 week each and eliminated thereafter. Additionally, every patient with risk constellation will receive cytomegalovirus (CMV) prophylaxis and prophylaxis against Pneumocystis carinii infection during the first 3 months after liver transplantation (Figure 1).

#### Follow-Up

Every patient will be followed up for 1 year after transplantation. The primary end-point will be at 30 days after transplantation (Steroid resistant acute rejection). During the first 30 days after transplantation there will be 9 visits where laboratory values (liver, renal and metabolic function, sirolimus trough levels), adverse events and rejection episodes will be recorded. Additionally there will be an ultrasound on day 1 after liver transplantation and on day 10 prior to the initiation of sirolimus to exclude hepatic artery thrombosis. Between day 30 and 1 year after liver transplantation the patient will be followed up to evaluate the long time outcome and secondary objectives of the trial. Table 2 gives an overview of planned examinations during follow-up.

**Table 2:** Follow-up and planned interventions for PATRON 07

	Day 0	Day 1	Day 2	Day 3	Day 4	Day 10	Day 14/15	Day 20/21	Day 25/26	Day 30	Month 3	Month 6	Month 12
**Medical History^1^**	X												

**Transplant Data**	X												

**eGFR**	X	X	X	X	X	X	X	X	X	X	X	X	X

**Laboratory^2^**	X	X	X	X	X	X	X	X	X	X	X	X	X

**Ultrasound^3^**	X	X				X					X	X	X

**Rejection^4^**		X	X	X	X	X	X	X	X	X	X	X	X

**AE**	X	X	X	X	X	X	X	X	X	X	X	X	X

**Sirolimus trough levels (4-10 ng/mL)**							X	X	X	X	X	X	X

1: focusing on liver disease, infectious history, concomitant renal disease

2: Na, K, creatinine, urea, GOT, GPT, AP, yGT, bilirubin, CHE, LDH, Quick, pTT, Hb, leucocytes, platelets, cholesterole and triglycerides (day 0 and 30)

3: Additional examinations in case of pathologic findings or indication.

4. laboratory signs of rejection (if suspected confirmed by biopsy)

### Clinical Sites

The study will be carried out at Regensburg University Medical Center.

### Safety aspects

All information and observations collected during the follow-up visits, as well as spontaneous reports from the patient, relatives or any other medical staff will be documented. Is an event serious (SAE) or suspected and unexpected (SUSAR) the particular authorities (BfArM [Bundesbehörde für Arzneimittel] and the Regensburg University Ethics Committee) will be informed. In case the safety and/or efficacy of the proposed treatment regimen will fail, the patient has to be switched to the center specific treatment regimen that potentially provides highest security with regard to safety and efficacy. Usually, this regimen will be based on a CNI. To evaluate the safety and efficacy of the proposed immunosuppressive regimen, a data safety monitoring board will be set up. After every third patient that finished the first 30 days period of the study, the monitoring board will receive data on efficacy and safety obtained from the patients. The board will consist of two physicians (one nephrologist and one hepatologist), who are both not involved in the study and independent from the conduct of the trial.

### Statistical Analysis and Number of patients needed

**Figure 2:**
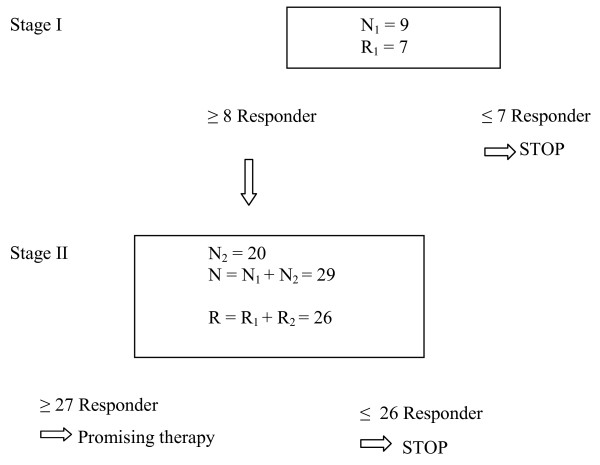
**Statistical flow-chart**. The flow chart shows the two-step model of the single-arm prospective trial. If there are more than 8 responders (patients that do not have steroid-resistant acute rejection within 30 days after transplantation.) after inclusion of 9 patients, there will be a second stepp with additional inclusion of 20 patients. If more than 27 patients do not show steroid resistant acute rejection, the proposed treatment regimen is promising.

The trial is designed on the basis of a critical review of the existing evident data on steroid- resistant acute rejection in liver transplantation and the use of de novo CNI-free immunosuppressive regimens. Steroid resistant acute rejection is the interesting parameter for the planned intervention since this is a threat for irreversible acute rejection and organ loss. A prospective uncontrolled two-step design was chosen for several reasons: there are hardly data on a de novo CNI-free immunosuppressive regimen. Therefore, we intended to design an uncontrolled pilot-trial, which is feasible for a single center, but is represented by a reasonable end-point. The two-step design offers this possibility with the inclusion of a first group of 9 patients, a consecutive evaluation and decision if the trial has to be terminated prematurely or if in the second step the hypothesis can be further confirmed by including additional 20 patients aiming at 29 patients in total [19]. This is a prospective, open-label, two-stage study. The primary endpoint is the incidence of steroid resistant acute rejection within the first 30 days after liver transplantation (LT). Based on Cochrane database and Medline database data including a total of 2.200 patients, the incidence of steroid resistant rejection within the first year after transplantation with the commonly used CNI-based and CNI-reduced protocols is 12.5% of all patients undergoing LT. This means that 87.5% of patients are without steroid resistant rejection (i.e., the response proportion is 87.5%). Based on this estimation a two-stage design [19] will be applied to test the following statistical hypothesis: *H_0_*: The true response probability *p *is less than the uninteresting level *p_0_*(*p *≤ *p_0_*). Versus *H_1_*: The true response probability *p *is at least the target level *p_1_*(*p *≥ *p_1_*). In this study, the uninteresting level will be defined as *p_0_*= 0.80, which means that 20% of our examined cohort would experience a steroid resistant early acute rejection within the first 30 days after LT. The target level will be defined as *p_1_*= 0.95, which means that 5% of our collective would experience a steroid resistant early rejection within the first 30 days. Under these assumptions, 9 patients will be enrolled in the first stage. After testing the immunosuppressive regimen on 9 patients in the first stage, the trial will be terminated if 7 or fewer respond to the therapy. If the trial goes to the second stage, an additional 20 patients will be enrolled. With a total of 29 patients, the following decisions will be made: If 26 or fewer responders are observed then H_0 _cannot be rejected. In this case, the immunosuppressive therapy is not promising with a high probability and is not worth to be tested in a greater trial for this indication. If 27 or more responders are observed, then H_0 _can be rejected. In this case, the immunosuppressive regimen is considered promising. If the therapy is actually not promising, there is a 0.049 probability of concluding that it is (the target significance level was set to 5%). If the therapy is actually promising, there is a 0.198 probability of concluding that it is not (the target for this value was set to 20%). The confirmatory analysis will be performed for the intent-to-treat population. Figure 2 shows a statistical flowchart for the trial.

### Trial organization, quality control and registration

The study was designed at Regensburg University Medical Center, Department of Surgery in cooperation with the Regensburg University Medical Center, Center for Clinical Studies. Quality assurance will be carried out in cooperation with the Clinical Transplantation Study Group of the Regensburg University Medical Center, Department of Surgery. The studies will be monitored by an independent monitor of the Clinical Transplantation Study Group. The studies will be performed in accordance with the Declaration of Helsinki in its current German version and the Good Clinical Practice/International Committee of Harmonization-guidelines (GCP/ICH). The study has been approved by the independent Regensburg University Ethical Review Board on November 16^th ^2007. Final approval for the trial by the BfArM has been obtained in November 2008 (EudraCT number 2007-003561-40). The start of the study was registered at the particular authority (Regierungsoberbehörde der Oberpfalz in Regensburg) on January 15^th ^2008. Additionally, it was registered under http://www.clinicaltrials.gov on January 15^th ^2008 (NCT00604357).

### Financial support

**PATRON07 **is funded by an internal liver transplant fund of the Department of Surgery at Regensburg University Medical Center.

### Current status

**PATRON07 **started to actively recruit in December 2008 and currently has 9 patients included, which indicates the first interim analysis. Data are currently cleaned and evaluated.

## Discussion

The **PATRON07**-study to establish CNI-free-"bottom-up" strategies in LT recipients with renal impairment prior to LT aims at minimizing IS in a "bottom-up" approach in contrast to classical "top-down" strategies aiming at improvement of renal impairment and infectious complications in a specific group of highly ill liver allograft recipients. **PATRON07 **is a prospective, non-controlled, two-stage study. Its objective is to evaluate the feasibility of a de novo CNI-free immunosuppressive regimen based on induction therapy with anti-CD25 monoclonal anti- body, mycophenolate mofetil (MMF/MPA), and mTOR-inhibition with sirolimus in patients with impaired renal function at the time of liver transplantation. The primary endpoint is defined as the incidence of steroid-resistant acute rejection within the first 30 days after liver transplantation. It reflects the early potential and feasibility of the proposed immunosuppressive regimen with regards to safety and efficacy. In a liver transplant setting an acute rejection can be controlled with steroid treatment and does not necessarily mean an irreversible damage to the liver. Therefore, classical endpoints in liver transplantation like renal outcome measures or graft and patient survival will be of interest once the completely new approach of bottom-up immunosuppression has been established.

Former studies evaluated the effect of the reversibility of CNI toxicity by introducing reduced or delayed CNI based regimens or by switching patients to other substances like mTOR-inhibitors or MMF in a "top-down"-fashion. Our intention with a de novo CNI free-"bottom-up" IS strategies is to generally avoid CNI toxicity in patients that already have impaired renal function a priori.

The study concept is designed as sequential concept performing 2 separate independent clinical trials. **PATRON07 **has to be performed as a first step since there is hardly published evidence for CNI-free de novo approaches with mTOR-inhibitors in LT collectives. The trial is designed as a prospective, uncontrolled, two-step design according to Simon [19]. This is a major advantage with regard to costs and feasibility of the trial. We intend to prove the safety and efficacy of a therapy regimen that has not been introduced in a prospective clinical setting in liver transplantation. Therefore, we first need safe and effective preliminary data from this pilot study to be able to offer it to a broader collective of patients in a prospective randomized trial focusing on a different renal-specific endpoint. With this design we found a suitable way to address a specific scientific clinical problem in a prospective setting, which is feasible for one single center without funding by a large pharmaceutical-company.

## Conclusion

We conclude, that if CNI-free-"bottom-up" IS strategies are safe and effective, this may be an innovative concept that could improve the patient short and long-time outcome with regards to renal function, infectious complications and avoidance of over-immunosuppression after LT. The results of **PATRON07 **may be the basis for a large multicenter RCT in patients with poor renal function at the time-point of LT.

## Conflict of Interests

The authors declare that they have no competing interests.

## Authors' contributions

AAS, MNS, EKG, AO and HJS developed the immunosuppressive strategy and protocols, wrote and revised the protocols and article

JS performed literature research and collected retrospective data from our liver transplantation patients to generate the hypothesis. Parts of the project will be his doctorial thesis.

JR is biostatistician. She developed the statistical model for PATRON07 and performed the sample size calculation for PATRON07.

SAF and ML are transplant surgeons and were involved in the discussion of the paper and reviewed the paper.

All authors read and approved the manuscript for publication.

## Pre-publication history

The pre-publication history for this paper can be accessed here:

http://www.biomedcentral.com/1471-2369/11/24/prepub
